# Comparison of zirconia degradation in dental implants and femoral balls: an X-ray diffraction and nanoindentation study

**DOI:** 10.1186/s40729-021-00383-2

**Published:** 2021-10-18

**Authors:** Javier Gil, José Angel Delgado-García-Menocal, Eugenio Velasco-Ortega, Begoña Bosch, Luis Delgado, Román Pérez-Antoñanzas, Mariano Fernández-Fairén

**Affiliations:** 1 Bioengineering Institute of Technology, Universitat Internacional de Catalunya, c/Josep Trueta s/n. 08195-Sant Cugat del Vallés, Barcelona, Spain; 2 School of Dentistry, Universitat Internacional de Catalunya, c/Josep Trueta s/n. 08195-Sant Cugat del Vallés, Barcelona, Spain; 3 Facultad de Odontología, Universidad de Sevilla, Sevilla, Spain

**Keywords:** Y-TZP, Fatigue and fracture, Degradation, Nanoindentation

## Abstract

**Background:**

New tetragonal zirconia polycrystal dental implants stabilized with yttria (Y-TZP) have appeared in the implantology market in the form of single piece or two-piece zircona implant system. These new type of implants improve the aesthetical properties compared to conventional commercially pure (c.p.) titanium used for implants, although the long term mechanical behavior of these new implants is not yet well known. In orthopaedics, the application of zirconia as femoral balls presented an important controversial use due to the premature fracture once implanted. Y-TZP dental implants can be affected by hydrothermal degradation and its behavior should be analysed to avoid a premature fracture. The scientific question behind the study is to analyse if the degradation mechanism observed in orthopaedics applications of Y-TZP is similar to that of Y-TZP for dental applications.

**Materials and methods:**

For this purpose, 30 original Y-TZP dental implants and 42 Y-TZP femoral balls fractured in vivo have been studied. Dental implants were submitted to an accelerated hydrothermal degradation to compare with the femoral balls fractured in vivo. Phase transformation as well as the mechanical behaviour of the degraded samples was studied by X ray diffraction and nanoindentation tests, respectively.

**Results:**

Results have shown that the fracture mechanism of dental implants does not resemble the mechanism observed in orthopaedic samples, presenting a good long-term behaviour.

**Conclusion:**

The results ensure the good performance of zirconia dental implants, because the degradation of the ceramic is very limited and does not affect the mechanical properties.

## Introduction

The development of. bioceramics for dental implantology is continuously increasing and has become an area of great interest in the field. Among the different bioceramics, the yttria-stabilized tetragonal zirconia (Y-TZP) is not only gaining interest as prosthesis but also as dental implant. The Y-TZP ceramics can be found as monolithic—implant and abutment in only one piece—which require extra efforts to optimize their structure, or can be composed of two separate articulated pieces, having the implant and the abutment in two separate pieces [1, 2].

At room temperature, zirconia ceramics present a tetragonal structure. The addition of yttria to the lattice (between 1.5 and 5% mol) metastabilizes the tetragonal phase at room temperature (over 1200 °C on pure zirconia). When the external stresses provoke a growing crevice in the material, the local stresses at the crack tip induce the transformation from tetragonal to monoclinic phase around the crack tip. This tetragonal-to-monoclinic transformation causes a volume growth of around 4%. This volume growth around the crack tip induces compressive stresses that tend to close the crack, increasing the fracture toughness of the material [3].

One of the main advantages of bioceramics is that these avoid issues related with corrosion, improving as well the wear resistance and presenting good aesthetic properties. However, ceramics are brittle and when used as ceramic implants, this present lower toughness than the c.p. Titanium. Table 1 shows the strength of the cp titanium and Y-TZP implants in different angles as specified in the ISO14801 standard [4, 5]. Values show that in the worse possible conditions (angle of 30°), the resistance of Y-TZP implants are higher than the behaviour of c.p. titanium implants. [6]. These properties have allowed the use of Y-TZP in the biomedical field, with special interest in load-transfer applications, including, dental implants.

**Table 1:** Mechanical resistance of the dental implants in different mechanical conditions according to the ISO14801 standard

Angle (°)	c.p.Titatium (grade 3)	Y-TZP (4.5% Y_2_O_3_)
	Maximum resistance (*N*)	Máximum resistance (*N*)
30	1378.7	2810.2
15	3046.7	8826.1
0	11,326.5	13,106.2

Flexural test at different angles

Biomedical grade zirconia has been previously used as femoral heads for orthopaedic hip implants. Previous results have shown that when the material is exposed to physiological conditions, a low temperature hydrothermal degradation takes places, which is also known as ageing. This ageing has produced significant amounts of femoral head fractures, leading to a minimal use of zirconia for this application [6]. The fracture of the femoral heads has been related to the interconnected microporosity that allows moisture to penetrate into the ceramic, facilitating ageing and the subsequent collapse of the material [7]. This type of failure mechanism is of particular interest for dental applications of zirconia. Nevertheless, several clinical studies revealed that the fracture of zirconia dental implants was rare [8]. In addition, others studies with zirconia abutments in intimate contact with the gingival tissue and moisture environment have shown that the failure of the implant was rare [9].

Hence, it is of great importance that the use of zirconia for dental implants takes into account this ageing phenomenon. It is relevant to analyse the aging process, allowing the characterisation the degraded layer and its influence on the mechanical properties to guarantee its use as dental implant. The purpose of this study was to quantify the mechanical response of Y-TZP dental implants previously degraded under different aging conditions and to compare the results with 42 femoral heads retrieved at different times after implantation. In this way, the aim is to compare the fractured femoral balls, of which we know the time in service, with zirconia dental implants that have been artificially aged.

## Materials and experimental methods

**Fig. 1:**
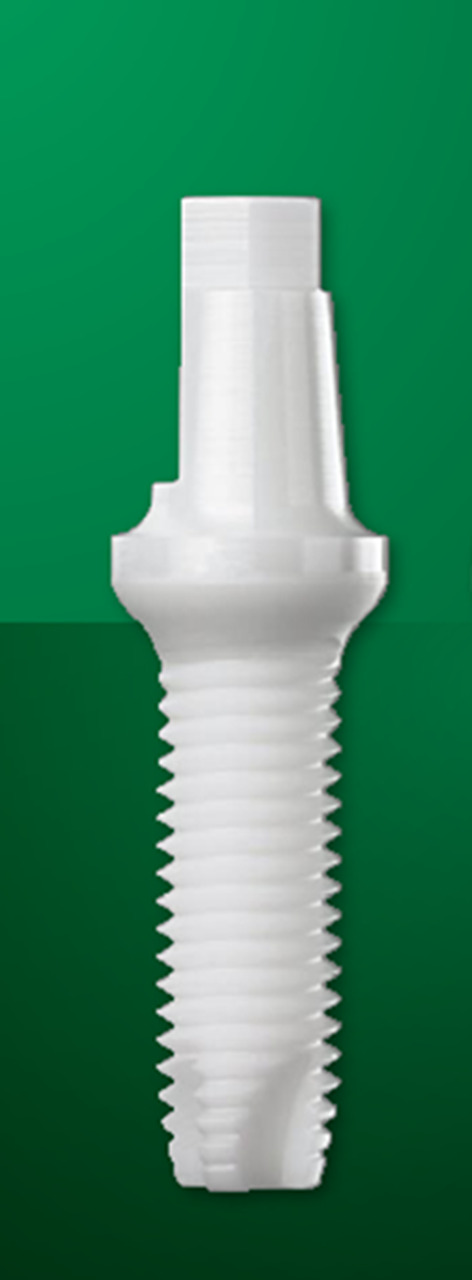
Zirconia one-piece dental implants studied

Thirty commercial biomedical grade yttria stabilized zirconia dental implants (Zlock3–411, Z-System, Konstanz, Germany) were acquired. The Zlock3-411 implants have a threaded length of 11 mm with a diameter of 4 mm and a diameter of the crest module of 6 mm. Implants are one-piece structure. Figure 1 shows the dental implants studied.

**Fig. 2:**
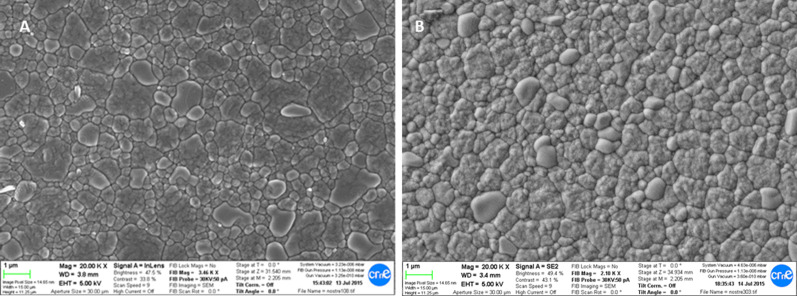
Microstructure of zircona observed by scanning electron microsocopy. **A** Dental implant. **B** Femoral head

Fifty femoral heads with a diameter of 28 mm were used in this study (Y-TZP A-BIO HIP® Thayngen, Switzerland). Eight heads were studied as control and 42 with different times in vivo. All the femoral heads were M-Type (for medium stem neck) and all stem replacements were made for titanium alloy Ti6Al4V. The chemical composition of the dental implants as well as of the femoral heads are shown in Table 2. In Fig. 2, the polygonal equiaxed grains in Y-TZP microstructure for dental implants (A) and for femoral heads (B). It can be observed the very similar grain size around 0.3 μm for both.

**Table 2:** Chemical composition of the commercial zirconia dental implants and femoral ball acquired for the studies (in % wt)

Oxides	Dental implant	Femoral ball
ZrO_2_	95.55	95.50
Y_2_O_3_	4.20	4.25
Al_2_O_3_	0.25	0.25

Dental implants and femoral balls presented only tetragonal phase in the control when the control samples were analyzed by XRD.

The Hospital Tres Torres collected 42 retrieved femoral heads at different in vivo times. The patients were 26 females and 16 males with ages from 56 to 81 years. The times of implantation ranged from 650 to 3116 days and the weight of the patients ranged from 44 to 84 kg. The reports of the clinical history are known.

The study was approved by the Research Ethical Committee of the Tres Torres Hospital reference number 1022014a and conducted according to the European Community guidelines for the care and use of laboratory animals (DE 86/609/CEE).

### Accelerated degradation process: low-temperature autoclave ageing

For the characterization, 10 implants were submitted to nanoindentation test after the degradation process, while the other 20 implants were submitted to mechanical tests after the different degradation times.

Each one of the Y-TZP Zlock3® dental implants of the first group were cutted to study the phases by X-ray diffraction. The cutting process was realised by a diamond disk to obtain 25 slices of 1 mm thickness and were mechanically polished with diamond paste from a suspension with a particle size of 30 up to 1 μm and finished with colloidal silica to give an average surface roughness of Ra < 5 nm. Finally, all samples were washed with acetone, methyl alcohol and dried at room temperature.

Twenty-eight samples were submitted to a degradation process conducted by means of an autoclave at 134ºC under 2 bar of pressure. The degradation times were 1, 2, 3, 4, 5, 7, 10, 11and 3100 h and were performed in triplicates [9]. Four samples were left as controls without performing any degradation procedure.

### X-ray diffraction

The martensitic transformation from tetragonal to monoclinic phase induced by the degradation was analysed by X-ray diffraction using a Bruker difractometer with Cu K_α_ of 1.54 Å. The different autoclave-degraded samples of the dental implants were analyzed, as well as samples of the femoral ball fractions, of which we know the live service times.

The voltage, intensity and step-size applied were 40 kV, 20 mA and 0.02°, respectively. To quantify the transformed phase fraction (*X*_m_), the following equations were employed [10]:

1 $$X_{{\text{m}}} = \frac{{I_{{{\text{m}}\left( {\overline{1}11} \right)}} + I_{{{\text{m}}\left( {111} \right)}} }}{{I_{{{\text{m}}\left( {{\overline{\text{1}}\text{11}}} \right)}} + I_{{{\text{m}}\left( {111} \right)}} + I_{{{\text{t}}\left( {101} \right)}} }},$$

where *I*_t_ and *I*_m_ represent the integrated intensity (area under peaks) of the tetragonal (101) and monoclinic (111) and (− 111) peaks. The volume of monoclinic phase (*V*_m_) is then given by

2 $$V_{{\text{m}}} = \frac{{1.311 \cdot X_{{\text{m}}} }}{{1 + 0.311 \cdot X_{{\text{m}}} }}.$$

### Nanoindentation test

Samples of each degradation time, as well as non degraded samples, were used for instrumented nanoindentation tests on an MTS XP System Nano Indenter. On each sample, nine monotonic tests were performed, in which the penetration depth was linearly increased up to the maximum depth of 1600 nm. The tests were conducted with a Berkovich tip with a measured radius of 750 nm. The elastic modulus and contact pressure values were evaluated with Continuous Stiffness Measurement (CSM) module [11]. The CSM module overlaps high frequency oscillations to the *P*–*h* curves, evaluating the elastic response from the unloading portion of such oscillations. All tests were conducted at 22 °C and the indenter was held in contact with the surface until the thermal drift was lower than 0.05 nm·s^−1^.

It is possible to know the thickness of the degraded layer by means of a model of thin layer properties that takes into account the contribution of the substrate to the mechanical response of the surface layer submitted to nanoindentation and proposes the next equation [12]:

3 $$\frac{1}{{E_{c}^{*} }} = \frac{2a}{{1 + \left( {{\raise0.5ex\hbox{$\scriptstyle {2t}$} \kern-0.1em/\kern-0.15em \lower0.25ex\hbox{$\scriptstyle {\pi a}$}}} \right)}}\left( {\frac{t}{{\pi a^{2} E_{f}^{*} }} + \frac{1}{{2aE_{s}^{*} }}} \right),$$

where: $E_{c}^{*}$ is the Apparent Young modulus obtained in the test.

$E_{f}^{*}$ is the Young modulus of the degraded layer.

$E_{s}^{*}$ is the substrate Young modulus. (210 GPa).

*a* is the contact radius corresponding to cylindrical indenter with the same contact area and it’s defined as: $A_{c} = \pi a^{2}$.

*t* is the thickness of the degraded layer.

From the nanoindentation tests together with Eq. (3), the film degraded as well as the Young modulus was determined in relation to the indentation depth for different layer thicknesses (*t*), choosing the value of *t* that produced a constant value across the indentation depth. In this case, a value of 165 GPa was chosen. Finally, it was possible to determine the degraded layer thickness.

For the determination of the degraded thickness, instead of nanoindentation, microindentation tests were performed by a Vickers and Knoop method with Matzsuzawa micro hardness) equipment (Tokyo, Japan using a load of 10 N (1Kgf) and an indentation time of 30 s.

### Mechanical tests

**Fig. 3:**
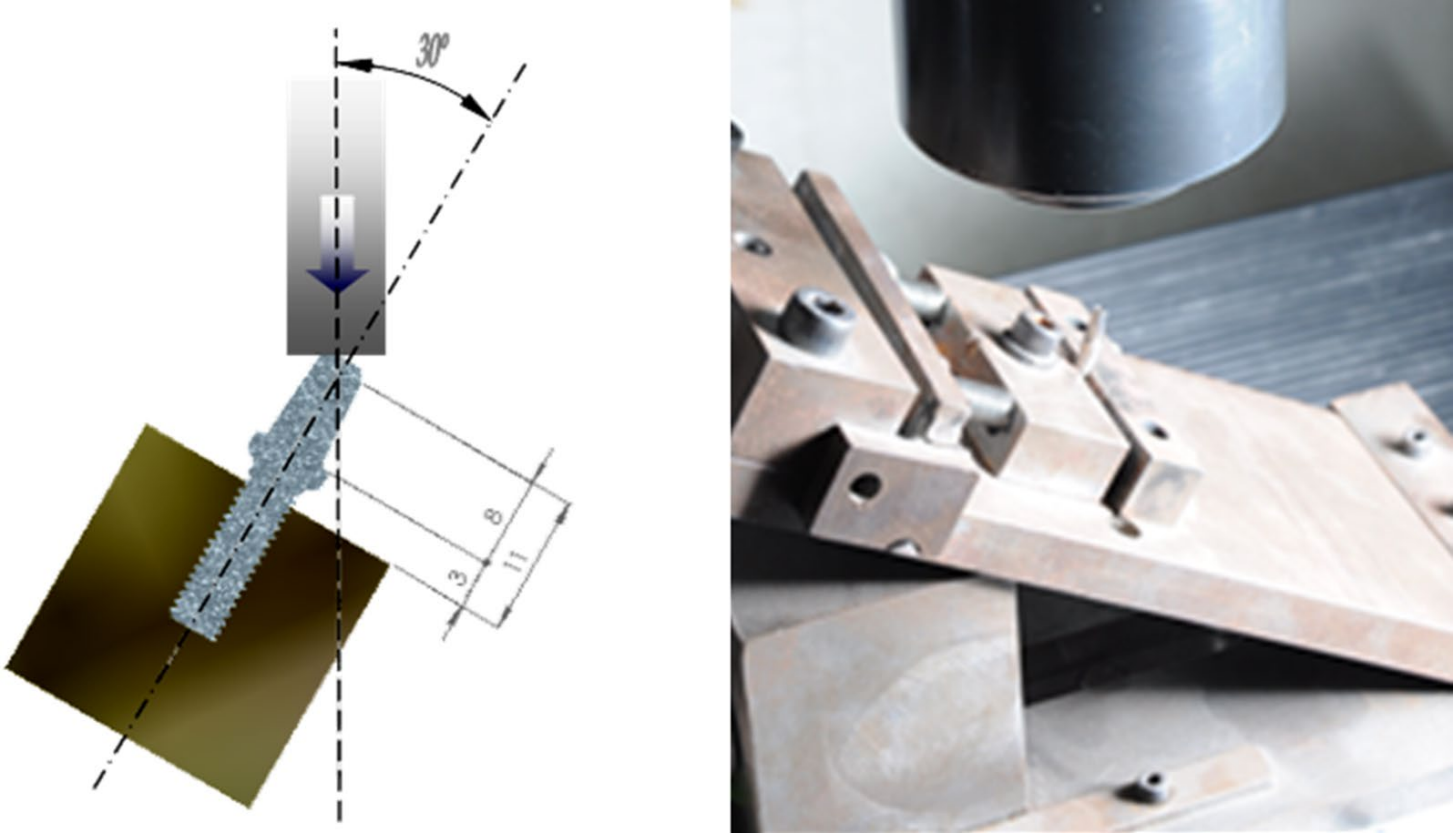
Mechanical tests system according to the ISO 14081

The mechanical tests were carried out following the ISO 14801 Standard [13]. Five Zlook3-411 were embedded in an acrylic resine Tecnovit 4071, Sulzer® (Switzerland) 3 mmover the nominal depth, simulating 3 mmof bone resorption, and placed in an angle of 30° with respect to the vertical axis of the implant (Fig. 3). The testing machine was a Bionix 858, MTS (Minnesota, USA) controlled by MTS Testworks 4 software. The test was performed under static conditions to determine the 30° flexure resistance of the implants.

### Statistics

Anova multiple-comparison Fisher’s tests were done with an appropriate software (Minitab ® 15.1.0.0, Minitab) to assess statistically significant differences between groups (*p* < 0.05).

## Results

**Fig. 4:**
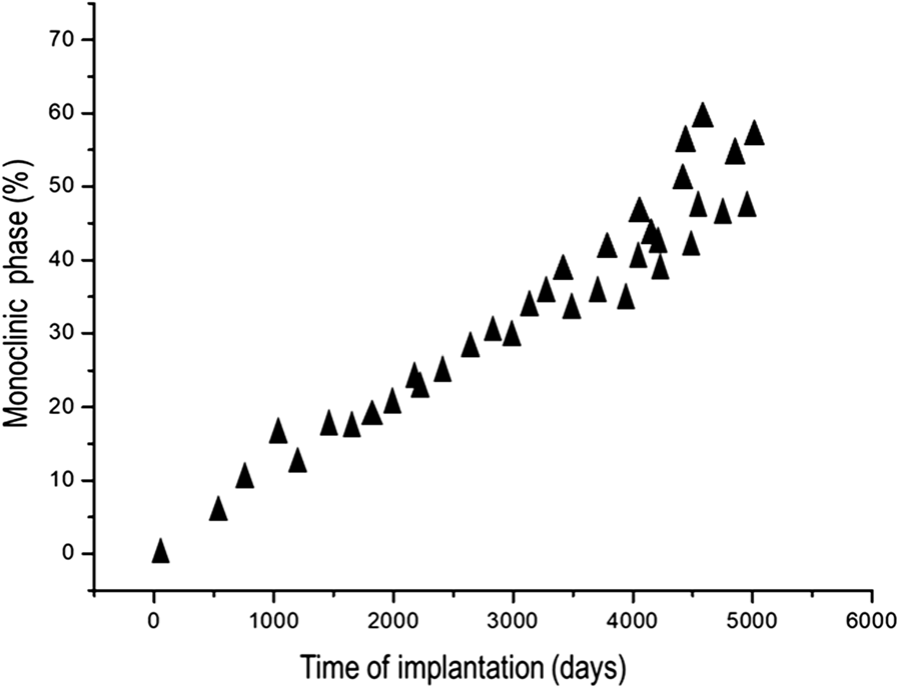
Monoclinic phase percentage present in the femoral heads in relation to the time of implantation

X-ray diffraction profiles of the degraded samples of zirconia femoral balls are shown in Fig. 4. Results showed a direct linear relationship between the time in service and the presence of the monoclinic phase, ranging up to 60% of monoclinic phase after 5000 days in vivo. The differences present statistical significance (*p* < 0.05) Furthermore, the control samples that were not placed in vivo and hence, not presenting the degradation process, showed a fully tetragonal pattern.

**Fig. 5:**
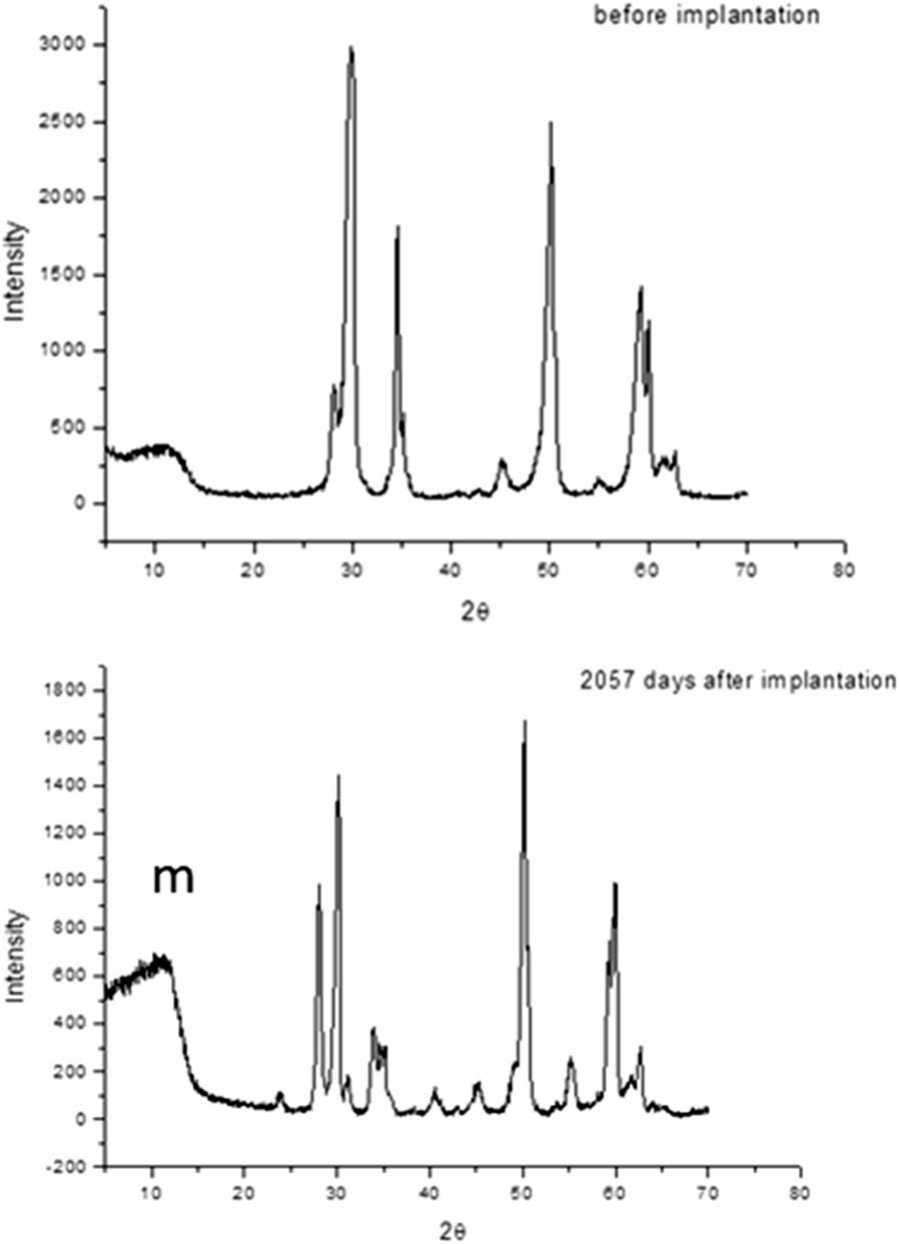
X-ray patterns of the zirconia femoral ball. Original and femoral ball retrieved after 2057 days in vivo, where the peak with m is characteristic of monoclinic phase

Figure 5 shows the X-ray patterns of the control zirconia femoral balls and the femoral balls implanted for 2500 days in vivo. The presence of the peak at the diffraction angle about 12° and the intensity changes are associated with the martensitic transformation from a tetragonal to monoclinic structure. The peak at the 12° angle complies with Bragg's law for the monoclinic phase and confirms the presence of this phase as a result of zirconia degradation. It is the product of the phase change of the tetragonal phase. X-ray analysis also allows us to quantify the percentage of transformation and, therefore, the degree of degradation. A notable increment of the phase transformed was shown as a function of the degradation times. Taking into account that the penetration of the X ray beam is around 1 μm, this technique is useful in the range of the beam penetration to detect the quantity of phase transformation.

**Fig. 6:**
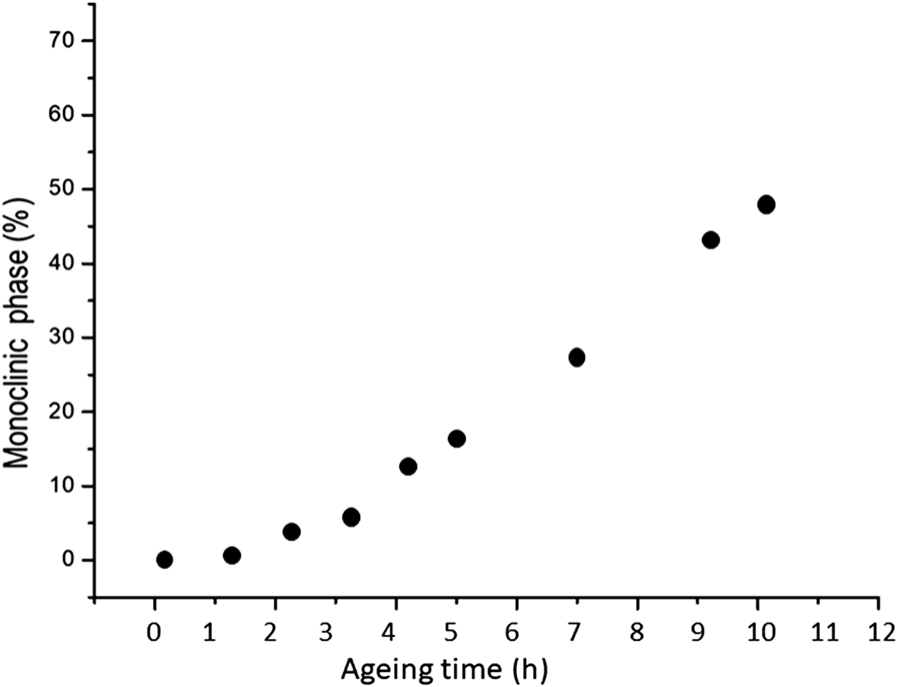
Monoclinic phase percentage presents in the dental implants in relation to the aging times

Figure 6 shows the increment of the monoclinic phase with the ageing time for the zirconia dental implants. Similar to the femoral balls, there was a direct linear increase of the monoclinic transformation with increased aging times. The results showed approximately 50% monoclinic transformation after 11 h of aging. These results present statistical significance with *p* < 0.05.

Table 3 shows constant hardness and elastic modulus values, obtained by nanoindentation for the zirconia dental implants. On the non-degraded samples, the average hardness value obtained was 17.1 ± 0.8 GPa with an elastic modulus of 245.8 ± 11,3 GPa. Conversely, a significant reduction of these properties was shown for all degraded samples. Table 4 shows the results regarding the femoral balls, showing the same tendency on decreasing the hardness, stiffness and elastic modulus as a consequence of the increase of the monoclinic phase. The differences of the results in all cases presented differences statistically significance (*p* < 0.05).

**Table 3:** Hardness, elastic modulus and degraded layer thickness of the different samples of dental implants in relation to the ageing times

Aging time (h)	Hardness (GPa)	Apparent elastic modulus (GPa) $E_{c}^{*}$	Degraded layer thickness (μm)
0	17.1 ± 0.8	245.8 ± 11.3	0
1	16.7 ± 0.6	244.9 ± 10.0	0.05 ± 0.01
2	16.2 ± 0.5	244.7 ± 11.2	0.12 ± 0.03
3	16.0 ± 0.3	242.3 ± 10.1	0.15 ± 0.03
4	15.8 ± 0.9	240.0 ± 10.9	0.21 ± 0.05
5	15.3 ± 1.0	236.2 ± 10.8	0.30 ± 0.07
7	15.1 ± 0.4	230.2 ± 12.9	0.70 ± 0.09
10	14.8 ± 0.7	229.8 ± 11.9	0.70 ± 0.10
11	14.6 ± 0.8	226.3 ± 10.7	0.72 ± 0.13

**Table 4:** Hardness, elastic modulus and degraded layer thickness of the different samples of femoral heads in relation to the in vivo times after implantation

In vivo time (h)	Hardness (GPa)	Average stiffness (N/mm)	Elastic modulus (GPa) *E*_c_^*^	Degraded layer thickness (μm)
0	16.7 ± 0.6	110 ± 9	247.8 ± 12.3	0
1124	15.8 ± 0.4	105 ± 7	243.9 ± 11.0	20 ± 8
2207	15.2 ± 0.5	100 ± 10	240.7 ± 10.1	160 ± 29
2507	14.5 ± 0.6	95 ± 9	238.6 ± 13.1	390 ± 50
3120	14.0 ± 0.8	96 ± 7	230.0 ± 10.0	709 ± 95
3789	13.3 ± 0.4	88 ± 9	224.1 ± 12.8	1300 ± 201
4123	13.1 ± 0.4	87 ± 4	216.2 ± 10.9	1800 ± 301
5120	12.0 ± 0.8	84 ± 7	207.8 ± 12.7	3807 ± 278
5350	12.2 ± 0.4	83 ± 9	202.3 ± 14.8	5099 ± 413
5888	11.8 ± 0.8	81 ± 8	201.8 ± 10.8	5308 ± 555

Table 5 shows the mechanical response of the Zlock3-411 dental implant to a monotonic 30° flexure test. Results show that the slope (stiffness) of the curve on the Y-TZP implants was 109.7 ± 8.78 N/mm. The behaviour of the Zlock3 implants was completely brittle and the maximum strength of Zlock3-411 implants was 767.3 ± 32.1 N. The most important result of the mechanical tests of the zirconia dental implants was that the results did not present statistical significant differences of strength in relation to the aging time. The results of the stiffness and maximum strength between 0 and 3100 h of degradation do not present differences statistically significance (*p* < 0.05).

**Table 5:** Mechanical properties of flexural strength at 30 °C of the zirconia dental implants at different ageing times

In vivo time (h)	Stiffness (N/mm)	Maximum strength (*N*)
0	107.1 ± 8.3	775 ± 8
1	109.6 ± 7.9	770 ± 7
2	112.2 ± 9.9	772 ± 9
3	116.0 ± 7.4	768 ± 7
4	118.2 ± 9.0	765 ± 8
5	104.3 ± 8.6	769 ± 9
7	103.2 ± 5.8	758 ± 5
10	110.2 ± 9.6	762 ± 9
11	106.2 ± 6.9	767 ± 6
3100	106.8 ± 5.2	766 ± 8

## Discussion

Martensitic transformation from a tetragonal to a monoclinic phase can be followed by X-ray diffraction during the hydrothermal degradation process which takes place when the zirconia stabilized with yttria is in contact with water. In the case of the hip prosthesis, this occurs in vivo as soon as the femoral balls are implanted. In the case of dental implants, this occurs to the zirconia dental implants after submitting the implants to an aging treatment [9–18]. In the present study, we studied fractured femoral balls retrieved from different patients and dental implants with different well controlled accelerated aging processes. The notable increment of the crystalline transformed phase as a function of the aging times is in agreement with the results of the retrieved in vivo femoral balls, showing that 1 h of aging process corresponds approximately to 50 h of in vivo implantation. The degradation function vs the time is linear in both cases [12–19].

A decrease of hardness and elastic modulus was present in all degraded samples, due to the phase transformation into monoclinic phase [19–21]. This reduction is attributed to a degradation process that starts on the surface and continues throughout the material in a very homogeneous manner. However, the degraded layer is higher in femoral balls than in dental implants. Table 5 shows that the degraded layer is practically constant in value near to 0.7 µm from the surface of the dental implants. This behaviour suggests a kinetic problem: there is no water diffusion process beyond 7 µm, and therefore, the process of hydrothermal degradation is stopped.

Consequently, the mechanical properties (Table 5) of the dental implants are not affected by the degradation time. This is due to the small and insignificant thickness of the dental implant that is affected by the transformation from tetragonal to monoclinic. That is to say the lixiviation of the yttrium from 0.7 µm does not occur, hence ensuring a dental implant with long term security. These results are in accordance with other authors, which found that the amount of monoclinic phase transformed on the surface of samples aged by autoclave was not sufficient to affect the inner part of the zirconia and, therefore, its mechanical properties [17–19]. The vacancies are occupied by OH^−^ ions on the surface and intergranularly diffuse into the interior of the specimen. Accordingly, the low-temperature degradation of ZrO_2_ was attributed to the annihilation of oxygen and yttrium vacancies by the OH − ions, and the grain boundaries were considered to play an important role in expanding the degradation. This diffusion is favoured by the wear subjected to stress.

A degradation study was carried out for 3100 h and it was observed that the values did not show statistically significant changes with respect to the values after 11 h. Therefore, we can assure that the degradation of zirconia in dental implants does not increase due to the lack of water diffusion, implying that the water does not exceed the degraded thickness and the phase transformation does not progress. This fact ensures that the dental implants do not undergo the degradative processes of the femoral balls. In the case of orthopaedic prostheses, the friction between components causes chipping of the ceramic which favors contact with water [8, 9, 20–24].

Nevertheless, the problem of hydrothermal degradation that has affected the Y-TZP Hip prostheses is different with respect to the dental implants. It can be explained, because the working mode of this material on a hip prosthesis and on a dental implant is completely different. In a hip prosthesis, the ball and the cup have a strong wear during each walking cycle and submitted to high stress [23–29]. When the water in contact with the Y-TZP components produce a lixiviation of yttrium and transforms the surface from tetragonal to monoclinic phase, it becomes rougher due to the volume change associated. The friction force provokes the fracture of the peak increasing body wear. The transformation from tetragonal to monoclinic phase growths to the inner of the femoral ball and the loss of mechanical properties takes place throughout the material [30–36]. This progression of the degradation is unstoppable because of the wear on the femoral ball. Ball fracture occurs when the mechanical properties of the mostly monoclinic ball cannot withstand the mechanical requirements to which it is subjected [36–39].

## Conclusions

A decrease in hardness and elastic modulus has been observed by means of nanoindentation as the monoclinic phase content increases (analysed by X-ray diffraction). It has been possible to verify that it only affects 0.7 µm from the surface in dental implants and this degradation does not significantly affect the mechanical properties of zirconia dental implants. Therefore, the long-term behaviour of dental implants are adequate from this point of view. However, the degradation depth of zirconia femoral balls from the surface is increasing with the loss of mechanical properties that make premature fractures of the prosthesis. This is due to the high tensions and the wear that is continuously occurring and favour the diffusion by grain boundaries. The slight degradation of zirconia in dental implants should be taken into account by clinicians. when placing this type of dental implants.

## Data Availability

Not applicable.

## Abbreviations

Y-TZP: Yttria tetragonal zirconia polycrystal
c.p. Ti: Commercially pure titanium
Ra: Average surface roughness
*X*_m_: The transformed phase fraction
*I*_t_: Integrated intensity (area under peaks) of the tetragonal (101)
*I*_m_: Integrated intensity (area under peaks) of the and monoclinic (111) and (− 111) peaks.
*V*_m_: Volume of monoclinic phase.
CSM: Continuous stiffness measurement
$E_{f}^{*}$: Is the Apparent Young modulus
$E_{f}^{*}$: Is the Young modulus of the degraded layer
$E_{s}^{*}$: Is the substrate Young modulus
*A*: Contact radius corresponding to cylindrical indenter with the same contact area
*A*_C_: Contact area
*t*: Thickness of the degraded layer
